# Supplementary material for: Two recipes for repelling hot water
Source: Nat Commun. 2019 Mar 29;10:1410. doi: 10.1038/s41467-019-09456-8 (PMC6440975; doi:10.1038/s41467-019-09456-8)
Supplement: Supplementary file 1 — Supplementary Information [file 41467_2019_9456_MOESM1_ESM.pdf]

Supplementary information

Two recipes for repelling hot water

T. Mouterde, P. Lecointre *et al.*

Supplementary figures

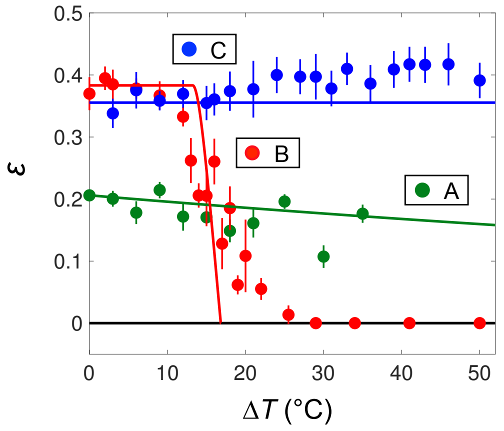

Supplementary Figure 1. Repellency for another drop radius. Coefficient of restitution ε as a function of the temperature difference ΔT for substrates A, B and C and water drops with radius $R \approx 1.1$ mm and velocity $V \approx 40\,\mathrm{cm\,s^{-1}}$. Coloured lines show the model (equation (5)) for the three regimes: $\tau > \tau_r$ (surface C), where no condensation adhesion occurs; $\tau < \tau_r$ (surface A), where condensation is immediate; $\tau \approx \tau_r$ (surface B), where the transition from bouncing to sticking is observed for $\Delta T \approx 17$°C. Error bars represent uncertainty of the measurement.

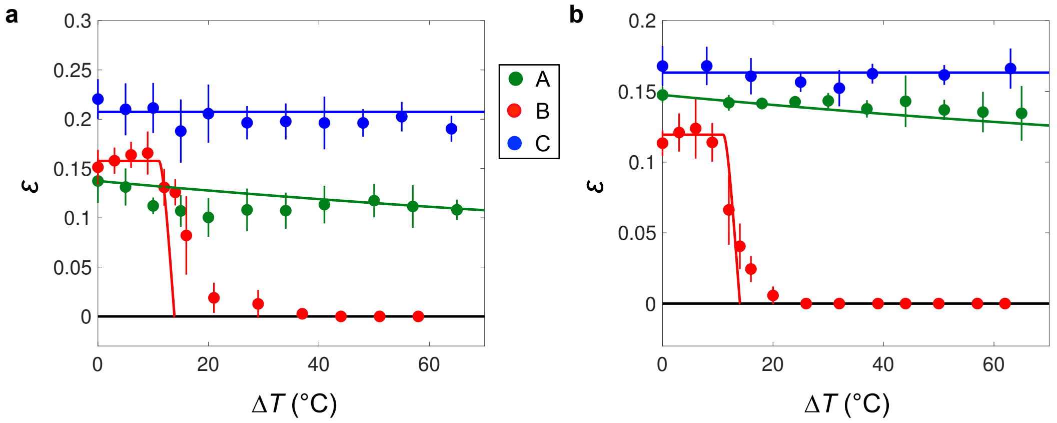

Supplementary Figure 2. Repellency at higher impact velocity. Coefficient of restitution ε as a function of the water/substrate temperature difference ΔT, for samples A, B and C, $R \approx 1.4$ mm and (**a**) $V \approx 59\ \mathrm{cm\,s^{-1}}$; (**b**) $V \approx 86\,\mathrm{cm\,s^{-1}}$. Solid lines show equation (5) and error bars represent uncertainty of the measurement.

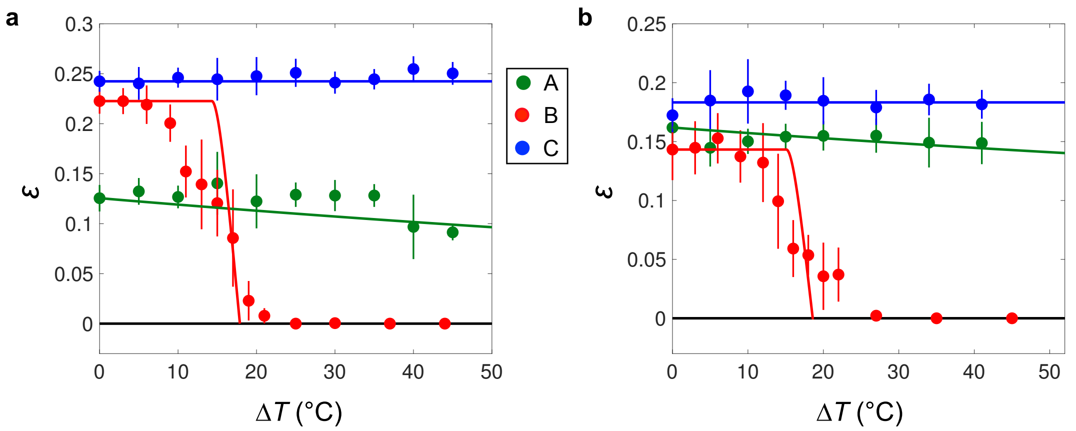

Supplementary Figure 3. Repellency at high impact velocity and smaller radius. Coefficient of restitution ε as a function of the water/substrate temperature difference ΔT, on samples A, B and C, for $R \approx 1.1$ mm and (**a**) $V \approx 59\ \mathrm{cm\,s^{-1}}$; (**b**) $V \approx 86\ \mathrm{cm\,s^{-1}}$. Solid lines show equation (5) and error bars represent uncertainty of the measurement.

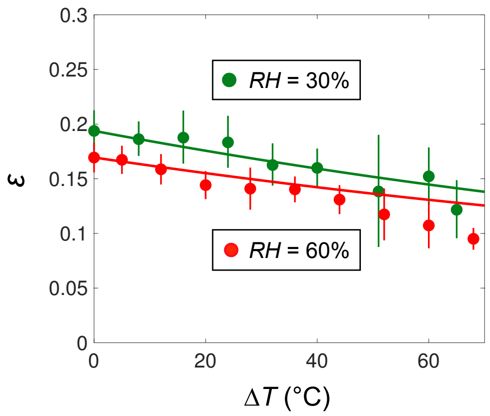

Supplementary Figure 4. Influence of hygrometry. Water drop coefficient of restitution ε as a function of the water/substrate temperature difference ΔT for two hygrometries, on sample A for $R \approx 1.4$ mm: $RH = 30\%$ and $V \approx 36\ \mathrm{cm\,s^{-1}}$ (green symbols); $RH = 60\%$ and $V \approx 40\ \mathrm{cm\,s^{-1}}$ (red symbols). Error bars represent uncertainty of the measurement.

Supplementary Discussion

We performed our experiments on samples A, B and C with a smaller water drop ($R = 1.10 \pm 0.05$ mm), at an impact velocity $V = 40 \pm 5\ \mathrm{cm\,s^{-1}}$ (Supplementary Figure 1). The characteristics of the impact are close to that reported in the Fig. 2d,e of the accompanying paper: the restitution coefficient ε of the shock is quite unsensitive to water temperature for sample C, it slightly decreases with sample A, and it evidences a sharp transition to sticking with sample B for $\Delta T \approx 17$°C. There again, data are convincingly fitted by equation (5) drawn

with solid lines. The adjustable parameter for the condensation time τ (in equation (1)) is the same as for the larger radius, *i.e.* $\alpha = 8$, which confirms the robustness of our model.

We also tested higher impact velocities, namely $V = 59 \pm 5$ cm s^{-1} and $V = 86 \pm 5$ cm s^{-1} – to be compared with V = 40 ± 5 cm s^{-1} in the accompanying paper. At smaller V, the contact time increases, which complicates the analysis; at larger V, water can splash, which also changes the physics. These experiments were carried out with two radii, $R = 1.10 \pm 0.05$ mm and $R = 1.40 \pm 0.05$ mm. As shown in Supplementary Figures 2 and 3, results are quite similar to that reported in the accompanying paper, and the model (equation (5), solid lines) convincingly fits the data at all explored values of V and R.

In order to quantify the influence of ambient humidity on bouncing, we performed an experiment with sample A (the sample having the smallest texture, that is, likely to be filled by small amounts of condensed water) at a hygrometry RH of both 30% and 60%, and for an atmospheric temperature $T_o = 20 \pm 1$°C. We plot in Supplementary Figure 4 the coefficient of restitution ε as a function of the excess temperature ΔT for $R = 1.4$ mm. The two series of data are found to be nearly superimposed, and both well described by the model (equation (5), drawn with solid lines), showing that repellency can resist significant variations of hygrometry. The slight shift between the two series of data arises from small differences in the impact velocity, that is, $V = 40 \pm 4$ cm s^{-1} for $RH = 60\%$ and $V = 36 \pm 4$ cm s^{-1} for $RH = 30\%$. A faster impact generates a less elastic shock (whatever the drop temperature), which explains the slight difference between both curves.

As shown in the Fig. 4 of the accompanying paper, our model allows us to predict a phase diagram for homothetic arrays of hydrophobic pillars, confirming the existence of two bounds in pillar heights h_1 and h_2 below and above which hot water always bounces. The two recipes for repelling hot water respectively correspond to $h < h_1$ and $h > h_2$. For “short” pillars, the probability $n(\Delta T)rp^2$ of finding a nucleus in a cell being small, h_1 is obtained by writing $\varepsilon = 0$ in equation (3) for $\Delta T_m = 75$°C (boiling point of water since the substrate temperature is around 25°C). This yields:

(6) $$h_1 = [\varepsilon_0 M V^2 / 4\pi\gamma R_m^2 r n(\Delta T_m)]^{1/2}$$

For typical values of the parameters, we expect that h_1 is on the order of 100 nm. Similarly, h_2 is obtained by writing $\varepsilon = 0$ in equation (5) for $\Delta T_m = 75°C$. For tall pillars, we have $n(\Delta T_m) > 1/rp^2$, which yields:

$$h_2 = [\tau_r D \Delta c_{sat}(\Delta T_m)/\alpha\rho]^{1/2}\,[1 - (\varepsilon_0 M V^2/4\pi\gamma R_m^2)^{1/2}]^{1/4} \quad (7)$$

For typical values of the parameters, h_2 is ~4 µm. We can further simplify the model by noticing that the correction to 1 in the bracket in equation (7) is ~0.13 – so that the first term only in the equation can be used for roughly estimating the value of h_2.

The two limits given by equations (6) and (7), drawn with a dotted line in Fig. 4, are analytical. We find in particular that h_1 depends on the coefficient of restitution ε_0 as $\varepsilon_0^{1/2}$, and that ε_0 only appears as a small correction in the expression of h_2. Both variations $h_1(\varepsilon_0)$ and $h_2(\varepsilon_0)$ are weak, which explains that taking a unique, average value $\varepsilon_0 = 0.2$ in the model allows us to draw a phase diagram (Fig. 4) valid for samples having slightly different ε_0.

Supplementary Note 1

We reported that the contact radius R_c can take the form $R_c \sim 2(RVt)^{1/2}$ at small time. This expression is valid for $t < 2R/V$, that is, around 7 ms in our situation – a time longer than the 5 ms needed for the drop to reach its maximum spreading radius ($R_c = R_m$). Besides, no rim is formed at maximal deformation, a consequence of the low Weber number ($We \approx 3$). This means that the thickness z of the pancake remains constant spatially so that the retraction speed can be correctly approximated by $(\gamma/\rho z)^{1/2}$.

Supplementary Note 2

We now detail the computation of the local contact time τ_L as a function of the distance from the impact point x. Denoting t_1 and t_2 ($t_1 < t_2$) as the solutions of the equation $R_c = x$, with $R_c(t) \approx 2(RVt)^{1/2} - (3\gamma/2\rho R)^{1/2} We^{1/4} t$, the local contact time is defined as $\tau_L = t_2 - t_1$. This equation is a second order equation in $t^{1/2}$. After expressing its two solutions $t_1^{1/2}$ and $t_2^{1/2}$, we calculate $\tau_L = t_2 - t_1$, which yields: $\tau_L = a(1-x/b)^{1/2}$ where $a = (8/3)\,(\rho R^3/\gamma)^{1/2}$ and $b = RWe^{1/4}$ denoting $We = \rho V^2 R/\gamma$ as the Weber number at impact.
