# Supplementary material for: Two recipes for repelling hot water
Source: Nat Commun. 2019 Mar 29;10:1410. doi: 10.1038/s41467-019-09456-8 (PMC6440975; doi:10.1038/s41467-019-09456-8)
Supplement: Supplementary file 2 — Description of Additional Supplementary Files [file 41467_2019_9456_MOESM2_ESM.pdf]

Supplementary Movie 1: Water drops (R = 1.4 mm and V = 40 cms-1) bouncing on materials A, A', B and C with respective pillar heights of about 100 nm, 200 nm, 1 µm and 10 µm. Both substrates and drops are at room temperature (ΔT = 0°C). Movies are slowed down 400 times.

Supplementary Movie 2: Hot water drops (R = 1.4 mm and V = 40 cms-1) impacting on materials A, A', B and C with respective pillar heights of about 100 nm, 200 nm, 1 µm and 10 µm. The temperature difference is set to ΔT = 21°C. Movies are slowed down 400 times.

Supplementary Movie 3: Hot water drops (R = 1.4 mm and V = 40 cms-1) impacting on materials A, A', B and C with respective pillar heights of about 100 nm, 200 nm, 1 µm and 10 µm. The temperature difference is set to ΔT = 40°C. Movies are slowed down 400 times.
